# Complete Genome Sequence of an Antimicrobial-Producing Bacillus velezensis Sam8H1 Isolate from the Makgadikgadi Saltpans of Botswana

**DOI:** 10.1128/MRA.00175-21

**Published:** 2021-04-29

**Authors:** Gorata Modikwe, Lokeshwaran Manoharan, Gomolemo Mabutho, Kebaneilwe Lebani, Lesedi Lebogang, Nerve Zhou

**Affiliations:** a Department of Biological Sciences and Biotechnology, Botswana International University of Science and Technology, Boseja, Palapye, Botswana; b Division of Occupational and Environmental Medicine, Department of Laboratory Medicine, Lund University, Lund, Sweden; c National Bioinformatics Infrastructure Sweden (NBIS), SciLifeLab, Lund University, Lund, Sweden; University of Maryland School of Medicine

## Abstract

The global antimicrobial drug resistance crisis requires urgency in searching for more effective broad-spectrum antimicrobial drugs. Here, we present a complete circular genome sequence and a plasmid of an antimicrobial-producing isolate, Bacillus velezensis strain Sam8H1, from the Makgadikgadi saltpans in Botswana. Bioinformatic analyses revealed 12 putative secondary metabolite biosynthetic gene clusters important for genome-guided drug discovery studies.

## ANNOUNCEMENT

**FIG 1:**
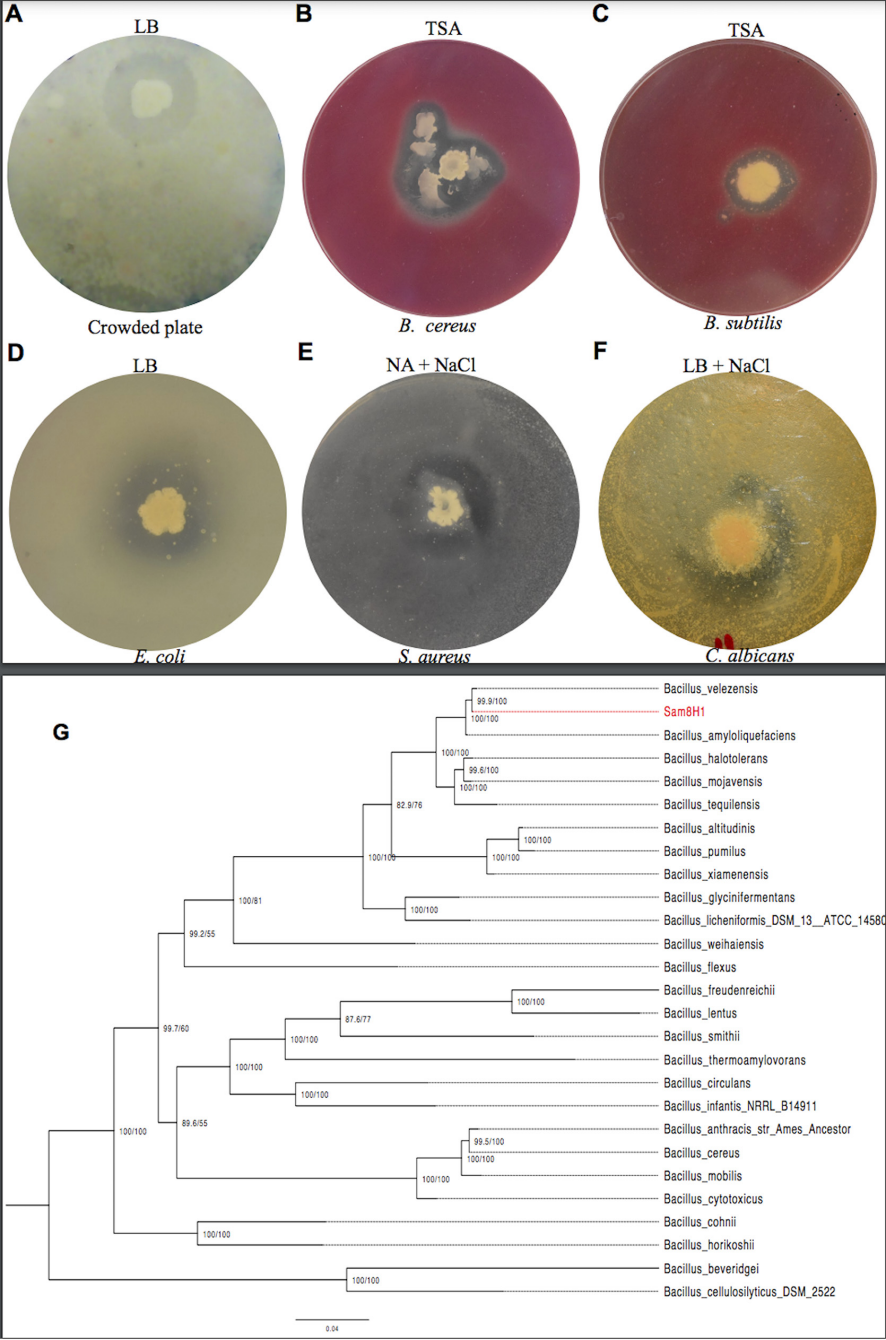
Antimicrobial activity of the isolate. Zone of inhibition after growth on Luria broth (LB) (A) and activity on Bacillus cereus (B) and B. subtilis (C) when grown on tryptic soy agar (TSA). (D) Activity on E. coli when grown on LB; (E) activity on S. aureus when grown on nutrient agar (NA) supplemented with NaCl; (F) activity on Candida albicans after growth on LB supplemented with NaCl. (G) Concatenated ribosomal protein tree constructed using IQ-TREE. As shown, Bacillus velezensis Sam8H1 is closely related to *B. amyloliquefaciens* and *B. velezensis* with very good branch support. Maximum likelihood tree based on the LG+F+I+G4 model with branch support from SH-aLRT and UF-boot.

The increasing burden of drug-resistant pathogenic microorganisms necessitated the search for novel antimicrobial producers inhabiting the extreme and underexploited Makgadikgadi saltpans of Botswana. Fifteen soil samples were collected and used to screen for antimicrobial-producing microorganisms using a crowded-plate technique described in reference 1. In brief, 0.5 g of each sample was resuspended in 1,000 μl of sterile distilled water, vortexed, and serially diluted 10-fold. Then, 100 μl was spread plated on Luria broth (LB) agar (10 g/liter tryptone, 10 g/liter NaCl, 5 g/liter yeast extract, and 15 g/liter agar [pH 7.0]). The plates were then incubated at 37°C for 24 to 48 h, and the zones of inhibition were examined. A single isolate from soil sample 8 and colony number H1, collected from 20°53′53.5″S, 25°49′09.4″E, exhibited a clear zone of inhibition (Fig. 1). The isolate was identified as Bacillus velezensis using 16S DNA PCR sequencing. Strain Sam8H1 belongs to the *B. velezensis* group with 99% similarity to *B. velezensis* CR-502^T^ (EZBioCloud database accession number AY603658), a species closely related to Bacillus subtilis and Bacillus amyloliquefaciens (2). The isolate was then screened for antimicrobial activity using an agar overlay assay as described in references 3 and 4 against the indicator strains Bacillus cereus (Carolina Biological 15-4872), B. subtilis (Carolina Biological 154921), Escherichia coli (ATCC 25922), Staphylococcus aureus (ATCC 25923), and Candida albicans (ATCC 10231) *in vitro* (Fig. 1 A to F).

For genomic DNA extraction, cells were grown overnight in LB at 37°C at 200 rpm, and DNA was extracted using a ZR soil microbe DNA isolation kit (Zymo Research, California) according to the manufacturer’s instructions. DNA was fragmented using G-tubes (Covaris) and end repaired to prepare a DNA library using a SMRTbell Express template prep kit v2.0 (Pacific Biosciences [PacBio], Menlo Park). All 20-kb libraries were size selected using the BluePippin system according to manufacturer’s recommendations (Sage Science, Beverly, MA, USA). Libraries were bound to the polymerase using a Sequel II binding kit v2.0 (PacBio, Menlo Park), followed by treatment with an enzyme cleanup kit (product number [PN] 101-843-100). The libraries were further size selected as described above using the BluePippin system and then sequenced using an RS II sequencer (PacBio).

A total of 35,761 HiFi circular consensus sequencing (CCS) reads were obtained for a total of ∼277 Mbp (70× coverage) of sequence with an *N*_50_ value of 7,714 bp. These reads were then trimmed, filtered (25,785 reads; ∼50× coverage), assembled, and, finally, circularized using the Canu v2.0 hierarchical assembly pipeline (5). Default parameters were used for all software unless otherwise specified. The total size of the genome after the assembly was 3.98 Mb (G+C content, 46.52%). Furthermore, it contained a circular plasmid of 148 kb (G+C content, 35.38%). A total of 4,041 open reading frames (ORFs) were predicted and annotated using PGAP (6); 192 of those genes were located on the plasmid. The genome contained 9 copies of 16S and 9 copies of 23S rRNA genes. Among the 16S rRNA genes, there were 5 identical copies that matched Bacillus velezensis with 100% identity (1,550/1,550 bp). For an in-depth analysis, 26 genomes of the *Bacillus* genus were downloaded from RefSeq (7), and a concatenated ribosomal protein-based phylogenomic tree (Fig. 1G) of these organisms was constructed. The sequences of all of the ribosomal proteins were aligned using MAFFT v3.710 (8), and then the alignments were cleaned using trimAl v1.2 (-gt 0.9, -cons 60) (9), resulting in 7,196 positions which were used to construct a phylogenetic tree using IQ-TREE v1.6.11 (-m TEST, -alrt 1000, -bb 1000) (10). Then, finally, the tree was visualized and annotated using FigTree v1.4.4 (11).

*In silico* analysis of the genome sequence using antiSMASH v5.1.2 (12) revealed that 12 putative biosynthetic gene clusters were relevant for producing antimicrobial compounds. The availability of this genome is important for further experimental studies toward the discovery of antimicrobial metabolites and provides insights into potential drug discovery.

### Data availability.

The complete genome sequence of Bacillus velezensis Sam8H1 can be accessed from the GenBank database under BioProject number PRJNA700378 and accession numbers CP069391.1 (genome) and CP069392.1 (plasmid). The PacBio CCS HiFi reads generated in this study can be found in the NCBI SRA under the accession number SRR13837247.
